# Smad2/4 Signaling Pathway Is Critical for Epidermal Langerhans Cell Repopulation Under Inflammatory Condition but Not Required for Their Homeostasis at Steady State

**DOI:** 10.3389/fimmu.2020.00912

**Published:** 2020-05-07

**Authors:** Linting Huang, Gui-Hua Li, Qian Yu, Yingping Xu, Steven Cvetkovski, Xuan Wang, Nirmal Parajuli, Imo Udo-Inyang, Daniel Kaplan, Li Zhou, Zhirong Yao, Qing-Sheng Mi

**Affiliations:** ^1^Department of Dermatology, Xinhua Hospital, Shanghai Jiao Tong University School of Medicine, Shanghai, China; ^2^Center for Cutaneous Biology and Immunology, Department of Dermatology, Henry Ford Health System, Detroit, MI, United States; ^3^Immunology Research Program, Henry Ford Cancer Institute, Henry Ford Health System, Detroit, MI, United States; ^4^Institute of Dermatology, Shanghai Jiao Tong University School of Medicine, Shanghai, China; ^5^Department of Dermatology, University of Pittsburgh, Pittsburgh, PA, United States; ^6^Department of Immunology, University of Pittsburgh, Pittsburgh, PA, United States; ^7^Department of Internal Medicine, Henry Ford Health System, Detroit, MI, United States

**Keywords:** Langerhans cells, Smad2, Smad3, Smad4, transforming growth factor-β1

## Abstract

Epidermal Langerhans cells (LCs) are skin-resident dendritic cells that are essential for the induction of skin immunity and tolerance. Transforming growth factor-β 1 (TGFβ1) is a crucial factor for LC maintenance and function. However, the underlying TGFβ1 signaling pathways remain unclear. Our previous research has shown that the TGFβ1/Smad3 signaling pathway does not impact LC homeostasis and maturation. In this study, we generated mice with conditional deletions of either individual Smad2, Smad4, or both Smad2 and Smad4 in the LC lineage or myeloid lineage, to further explore the impact of TGFβ1/Smad signaling pathways on LCs. We found that interruption of Smad2 or Smad4 individually or simultaneously in the LC lineage did not significantly impact the maintenance, maturation, antigen uptake, and migration of LCs *in vivo* or *in vitro* during steady state. However, the interruption of both Smad2 and Smad4 pathways in the myeloid lineage led to a dramatic inhibition of bone marrow-derived LCs in the inflammatory state. Overall, our data suggest that canonical TGFβ1/Smad2/4 signaling pathways are dispensable for epidermal LC homeostasis and maturation at steady state, but are critical for the long-term LC repopulation directly originating from the bone marrow in the inflammatory state.

## Introduction

Epidermal Langerhans cells (LCs) functionally serve as a unique subset of skin-resident dendritic cells (DCs), although they are also classified as a specialized macrophage subset in skin based on their developmental relationship like tissue resident macrophage derived from embryonic precursors (1). LCs represent the first antigen-presenting cells in the skin that encounter foreign antigens and take part in a variety of immunological processes. “Classical” epidermal LCs, characterized by the expression of a C-type lectin receptor known as Langerin (CD207), are defined as CD45^+^ MHCII^+^ Langerin^+^ cells (2–5). LCs phagocytize epidermal detritus with their dendritic protrusions and eventually present these antigens on the cell surface with an MHCII molecule (6). Following cell activation by various stimuli, LCs have increased expression of costimulatory markers CD80 and CD86, decreased expression of epithelial cell adhesion molecule (EpCAM) and E-cadherin, and acquire a migratory phenotype. These changes result in LC migration to the regional draining lymph nodes (LNs), where the LCs interact with naïve T cells to induce immunity or tolerance (3, 7). At steady state, LCs self-renew, while under the inflammatory state, such as ultraviolet (UV) light exposure-induced inflammation, there are two waves of LC repopulation in the skin (8). The first wave involves fast “short-term” LCs that are derived from peripheral blood Gr-1^hi^ monocytes and express low levels of Langerin, while the second wave generates “long-term” LCs that are derived from bone marrow (BM) and express high levels of Langerin (8). Despite recent research advances, the specific underlying mechanisms that precisely regulate LC development and function are still not fully understood.

Transforming growth factor-β (TGFβ) is a key cytokine for the differentiation and maintenance of LCs. The regulatory roles of TGFβ1 include promoting LC development, inhibiting LC maturation and migration to draining LNs (9), and regulating LC repopulation during inflammation (8). LCs are absent in mice that lack TGFβ1 due to failure of LC differentiation, survival, or both (9–11). Although TGFβ1 is expressed by both keratinocytes and LCs, an autocrine source of TGFβ1 is required for LC development (9). However, the underlying mechanisms of TGFβ1 signaling in LCs remain elusive. In the classical TGFβ1 signaling pathway, TGFβ1 signals through TGFβ receptor 2 (TGFβR2) and TGFβ receptor 1 (TGFβR1). Subsequently, these receptors activate two structurally-similar proteins, Smad2 and Smad3, which leads to the formation of a heterotrimeric complex with a common Smad4 that translocates to the nucleus to regulate TGFβ1 target genes (12, 13). Some Smad-independent pathways, such as Erk, JNK, and p38 MAPK kinase, also mediate TGFβ1 signaling (13, 14). Given the ubiquitous influence of TGFβR/Smad signaling on immune regulation (13, 15), we sought to investigate how Smad2/3/4 proteins in the TGFβ1 pathway regulate LC development.

Due to the embryonic lethality of conventional Smad2 and Smad4 knockout mice, we previously used conventional Smad3 knockout (Smad3KO) mice to study the role of the TGFβR/Smad signaling pathway in LC development. We found that the TGFβR/Smad3 signaling pathway was inessential for LC development (16). Therefore, it raises the possibility that Smad2 or Smad4 may be involved in TGFβ1-mediated LC development. Human Langerin^Cre^ (hLan^Cre^) and Csf1r^Cre^ reporter mice are reliable tools to study LCs in mice. hLan^Cre^ induces LC-exclusive gene deletion after birth, while Csf1r^Cre^ induces gene deletion in monocytes and macrophages in addition to LCs (4, 9). Here, we generated the mice with conditional deletions of either Smad2 or Smad4, or both Smad2 and Smad4 in LCs, mediated by either hLan^Cre^ or Csf1r^Cre^. We compared the frequency and function of LCs between knockout (KO) and wild type (WT) littermates. Similar to our previous observations in Smad3KO mice, the Smad2/4 signaling pathway was not required for LC maintenance, maturation, antigen uptake, or migration *in vivo* or *in vitro* in the steady state. We found that Smad2/4 signaling was involved in BM-derived LC replenishment after UV exposure. Therefore, our results further demonstrate that TGFβ1 regulates LC homeostasis through a Smad4-independent pathway during steady state and suggest that TGFβ1 regulates LC repopulation through a Smad4-dependent pathway during the inflammatory state.

## Materials and Methods

### Mice

Csf1r^Cre^ mice (#021024) and Smad4^fl/fl^ mice (#017462) were purchased from Jackson Laboratory. The Smad2^fl/fl^ mice were kind gifts from Dr. Daniel Bernard at McGill University. Human Langerin^Cre^ mice (9) were reported previously. All mice were backcrossed to C57BL/6J genetic background for at least six generations. The experiments were conducted in 7- to 12-week-old mice (both sexes) unless otherwise indicated. The mice were bred and maintained in a pathogen-free facility at the Laboratory Animal Services Center of Henry Ford Health System. Handling of mice and experimental protocols were in accordance with the Institutional Animal Care and Use Committee of Henry Ford Health System.

### Epidermal Langerhans Cells Suspension Preparations

Mouse skin was harvested and processed as previously described (17). Briefly, the separated epidermal sheet was first incubated in 0.25% Dispase (Gibco, Japan) for 1 h at 37°C. Next, the epidermal sheet was briefly digested in RPMI (HyClone, Logan) containing 10% FBS (ATLANTA biologicals, Flowery Branch) and 0.01% DNase I (Worthington, Lakewood). The sample was filtered through a 40 μm mesh, and epidermal single cells were collected for flow cytometry.

### Lymph Node Suspension Preparations

Lymph nodes (LNs) were harvested and incubated in PBS containing 3% FBS, 1 mg/mL collagenase D (Roche Diagnostics, Germany), and 100 U/mL DNase I (Worthington, Lakewood) for 20 min at 37°C. The sample was filtered through a 40 μm mesh and LN single cells were collected for flow cytometry.

### LC Antigen Uptake *in vitro*

Dextran-Fluorescein isothiocyanate isomer I (FITC) (Life Technologies, NY) was added into the epidermal suspension cells to a final concentration of 0.025%. The epidermal cells were shaken and incubated for 45 min at either 4 or 37°C. Following incubation, the epidermal cells were collected for flow cytometry analysis (18).

### LC Culture *in vitro*

Freshly-isolated epidermal cells were suspended in RPMI containing 10% FBS, 5.5 × 10^−5^ 2-Mercaptoethanol (Gibco, Grand Island), 0.02 M HEPES buffer (Corning, Manassas), 1X MEM Non-essential Amino Acids (Gibco), 1 mM Sodium Pyruvate (Gibco), 100 U/mL Penicillin, and 100 μg/mL Streptomycin, then were incubated for 72 h at 37°C. Following incubation, the cells were collected for flow cytometry analysis.

### LC Migration and Phagocytosis *in vivo*

Mouse ear (10 μL) or belly (200 μL) was painted with 5 mg/mL FITC (Sigma, St. Louis) in acetone/dibutyl phthalate (1:1). After 24 h of treatment, the skin-draining LNs were collected for flow cytometry analyses (18).

### Flow Cytometry

Cells were resuspended and mixed with anti-CD16/32 (clone 2.4G2) for 10 min at 4°C, then stained with the following antibodies: CD80 (16-10A1), CD86 (GL-1), Langerin (4C7), MHCII (M5/114.15.2), CD45 (30-F11), and EpCAM (G8.8). All antibodies used were from Invitrogen, eBioscience, Tonbo, BD Biosciences, or BioLegend. The cells were acquired or sorted using a Becton Dickinson FACSAria II flow cytometer (BD Biosciences, San Jose). Data were analyzed in live cells using the FlowJo software after gating out double cells (Tree Star, Ashland).

### RNA Extraction and qRT-PCR

RNA was isolated using the GenElute™ Total RNA Purification Kit (Sigma). qRT-PCR was performed using the QuantStudio 7 Flex Real-Time PCR System. Quantitative gene expression data were normalized to the expression of GAPDH. Primers used are as below: GAPDH F5′- GGTGAAGGTCGGTGTGAACG−3′ R5′- TGTAGACCATGTAGTTGAGGTCA−3′; Smad2 F5′- CCCACTCCATTCCAGAAAAC−3′ R5′- GAGCCTGTGTCCATACTTTG−3′; Smad4 F5′- CCAGGATGGTGGACTATGAAAT−3′ R5′- GCAGCAAACACATCTCTCAAC -3′.

### Immunofluorescence

Mouse ear tissue was first floated on 20 mM EDTA (AMRESCO, Ohio) overnight at 4°C. Then, the epidermal sheets were detached from the dermis and fixed in acetone for 15 min at −20°C and placed on slides. The samples were blocked by 1% anti-CD16/32 (clone 2.4G2) in 3% BSA and stained with 1% MHCII (M5/114.15.2). The slides were examined with an Olympus FSX100 microscope.

### LC Replenishment After Skin Inflammation

The mice were anesthetized, and the shaved back was exposed to ultraviolet (UV) light for 15 min (wavelength: 254 nm; voltage: 8 W; source: 38 cm) as previously described (18). The mice were sacrificed at day 5 or day 20 post-UV treatment for LC replenishment analysis.

### Statistical Analysis

Statistical analysis was performed with Prism 7.0 (GraphPad, La Jolla, Calif) and SPSS (IBM, NY). Data were analyzed using the two-tailed Student's *t*-test when variances were equal; otherwise, the unpaired *t*-test with Welch correction was used. A *p*-value of <0.05 was considered to be statistically significant.

## Results

### Smad2 Is Not Required for LC Maintenance

**Figure 1:**
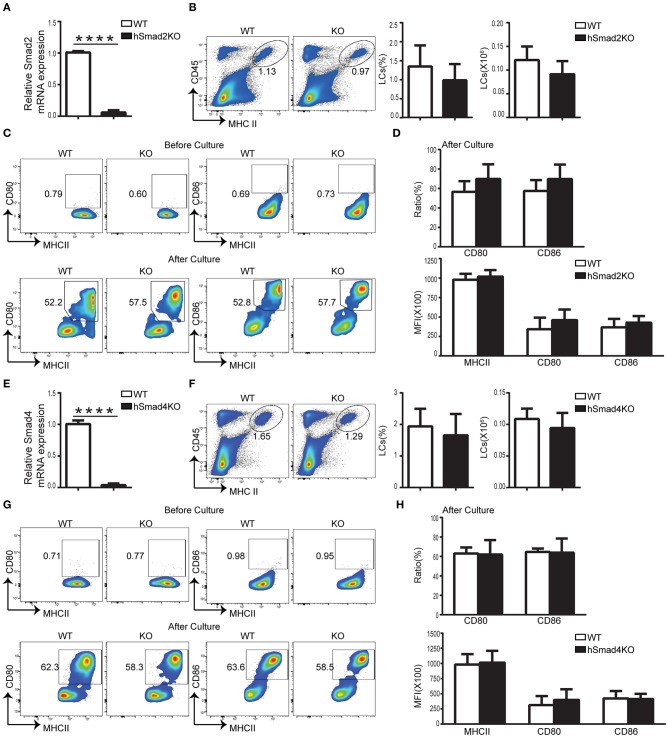
Smad2 or Smad4 deficiency does not impact LC development or maturation. **(A–D)** In hSmad2KO and WT mice: **(A)** Expression of Smad2 in sorted LCs (CD45^+^ MHCII^+^) from hSmad2KO and WT mice by qRT-PCR (*n* = 6, ****P < 0.0001). Frequencies and number of LCs (CD45^+^ MHCII^+^) **(B)** and related maturation markers **(C-top)** from hSmad2KO mice and WT littermates at steady state (*n* = 8, *P* > 0.2). Flow cytometric analyses of LCs **(C-bottom)**, the frequency of MHCII, CD80 and CD86 and their expression of MFI (median fluorescence intensity) **(D)** after 72 h of *in vitro* culture were shown (*n* = 6–11, *P* > 0.1). **(E–H)** In hSmad4KO and WT mice: **(E)** Expression of Smad4 in sorted LCs from hSmad4KO and WT mice by qRT-PCR (*n* = 6, ****P < 0.0001). **(F,G)** Frequency, number **(F)** and maturation **(G-top)** of LCs from hSmad4KO mice and their WT littermates at steady state (*n* = 10, *P* > 0.3). Flow cytometric analyses of LCs after 72 h of *in vitro* culture **(G-bottom)**. The frequency of MHCII, CD80 and CD86 and their expression (MFI) **(H)** after *in vitro* culture were shown (*n* = 6–16, *P* > 0.3). Data were shown as mean ± SD.

We previously reported that TGFβR/Smad3 signaling pathway was inessential for LC development (16). To investigate the role of Smad2 in LC regulation, we crossed Smad2^fl/fl^ mice with hLan^Cre^ mice (19) to generate mice with LC-exclusive Smad2 deletion after birth. Expression of Smad2 in epidermal LCs from hLan^Cre^ Smad2^fl/fl^ mice (hSmad2KO) was dramatically reduced compared to WT littermates; this was confirmed by quantitative RT-PCR (qRT-PCR) (Figure 1A). LCs are the only epidermal CD45^+^ MHCII^+^ population in the epidermis at the steady state. As shown in Figure 1B, there was no significant alteration in the ratio or number of epidermal LCs (CD45^+^ MHCII^+^) between WT and hSmad2KO mice; this was analyzed by flow cytometry. Therefore, the specific loss of Smad2 in the LCs had no significant effect on LC maintenance.

### Smad2 Deficiency Does Not Hamper LC Maturation

During cell maturation, LCs have increased expression of MHCII and cell membrane costimulatory molecules, such as CD80 and CD86. Prior research has shown that LC-specific deletion of either TGFβ1, TGFβR1, or TGFβR2 results in spontaneous LC maturation and increased expression of CD80 and CD86 (19, 20). Given these research findings, we next examined whether Smad2 is necessary for maintaining LC immaturity. As shown in Figure 1C, the expression of MHCII, CD80, and CD86 in LCs were unaltered in hSmad2KO mice compared to WT mice at steady state. This finding contrasts previous observation that LCs spontaneously matured in the mice with the deletion of TGFβ1 or its receptors (19, 20). Similar to WT mice, LCs from KO mice had increased expression of MHCII, CD80, and CD86 after 72 h of *in vitro* culture, and there was no significant difference in the frequency and MFI (median fluorescence intensity) of MHCII, CD80, or CD86 in LCs between KO and WT mice, respectively, after 72 h of *in vitro* culture (Figures 1C,D). These observations indicate that, like Smad3 (16), Smad2 is not essential to maintain LC immaturity during steady state or for LC maturation upon stimulation.

### Smad4 Is Not Required for LC Homeostasis or Maturation at Steady State

Phosphorylated Smad2 and Smad3 lead to the formation of a heterotrimeric complex with Smad4 that translocates to the nucleus to regulate TGFβ1 target genes (12, 13). To further investigate the role of the TGFβ1/Smad pathway, especially for Smad4 in LC development, hLan^Cre^ mice were crossed with Smad4^fl/fl^ mice to generate LC-specific Smad4 KO mice (hSmad4KO). The deletion of Smad4 in epidermal LCs was confirmed by qRT-PCR (Figure 1E). As shown in Figure 1F, there was no significant difference in the epidermal LC ratio or the LC number between hSmad4KO and WT littermates. Therefore, Smad4 is not required for LC homeostasis. Next, we examined the expression of CD80 and CD86 in LCs at the steady state, and after 72 h of *in vitro* culture (Figures 1G,H), there was no significant difference between hSmad4KO mice and WT littermates under both conditions. Therefore, our current data and the previous data from Smad3KO mice (16) further indicate that the Smad2/3/4 pathway is not essential for LC homeostasis or maturation.

### Smad2 Is Dispensable for LC Phagocytosis and Migration

**Figure 2:**
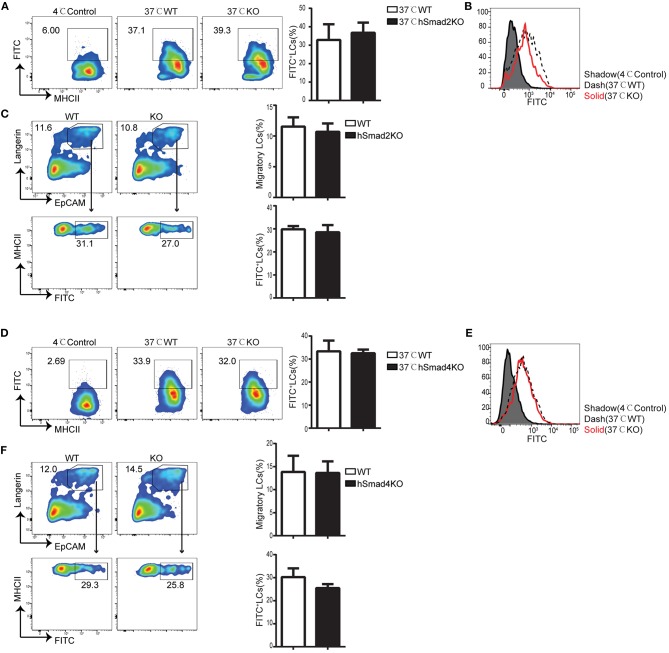
Smad2 or Smad4 deficiency does not interrupt LC phagocytosis and migration. **(A–C)** In hSmad2KO and WT mice: **(A)** Representative flow cytometry analysis (left) and the frequency (right) of FITC^+^ LCs (MHCII^+^ FITC^+^ gated on CD45^+^ cells) after incubation with Dextran-FITC for 45 min at 37 or 4°C (as control) in hSmad2KO and WT mice (*n* = 6, *P* >0.5); **(B)** FITC MFI in live LCs was shown; **(C)** Draining lymph nodes (LNs) were collected 24 h after FITC treatment on skin, flow cytometry plots (left) and the frequency (right) of migratory LCs (Langerin^+^ EpCAM^+^ on pre-gated CD45^+^ MHCII^hi^ cells) (top) and LCs captured antigen (MHCII^+^ FITC^+^ on pre-gated CD45^+^MHCII^+^Langerin^+^EpCAM^+^ cells)(bottom) were shown (*n* = 6, *P* > 0.5). Data were shown as mean ± SD. **(D–F)** In hSmad4KO and WT mice: **(D)** Representative flow cytometry analysis (left) and the frequency (right) of FITC^+^ LCs (MHCII^+^ FITC^+^ gated on CD45^+^ cells) after incubation with Dextran-FITC for 45 min at 37 or 4°C (as control) in hSmad4KO and WT mice (*n* = 7, *P* > 0.5); **(E)** FITC MFI in live LCs were shown **(F)** Draining LNs were collected 24 h after FITC treatment, flow cytometry plots (left) and the frequency (right) of migratory LCs (Langerin^+^ EpCAM^+^ on pre-gated CD45^+^ MHCII^hi^ cells) (top) and LCs captured antigen (MHCII^+^ FITC^+^ on pre-gated CD45^+^MHCII^+^Langerin^+^EpCAM^+^ cells)(bottom) were shown (*n* = 6, *P* > 0.5). Data were shown as mean ± SD.

Epidermal LCs acquire and process antigens and migrate to skin-draining LNs. Then, the LCs present the antigens to T cells and mediate antigen-specific immunity or immune tolerance (21–23). To evaluate the role of Smad2 in LC phagocytosis, we incubated freshly-isolated epidermal cells from hSmad2KO and WT mice with Dextran-Fluorescein isothiocyanate isomer I (FITC) for 45 min at 37 or 4°C (control). The LCs labeled with Dextran-FITC (CD45^+^ MHCII^+^ FITC^+^) were defined to have effective phagocytosis. The ratio and MFI of FITC^+^ LCs were comparable between hSmad2KO and WT mice (Figures 2A,B). Next, we further investigated the role of Smad2 in LC antigen capture and migration *in vivo* by FITC painting (24). We analyzed the frequency of migratory FITC^+^ LCs (CD45^+^ MHCII^+^ Langerin^+^ EpCAM^+^ FITC^+^) in skin-draining LNs and observed no significant difference between hSmad2KO and WT mice (Figure 2C). Therefore, Smad2 deficiency has no significant impact on LC antigen uptake or migration. This finding is similar to the previous observation with Smad3 deficiency (16).

### Smad4 Is Not Essential for LC Phagocytosis and Migration

We next examined the function of Smad4 in LC phagocytosis and migration. The freshly-isolated epidermal cells from hSmad4KO and WT mice were incubated with Dextran-FITC, and we found that there was no significant difference in the frequency or MFI of FITC^+^ LCs between hSmad4KO mice and WT littermates (Figures 2D,E). Therefore, LC-specific Smad4 deletion had no significant effect on LC antigen-capture capabilities *in vitro*. We next examined the potential impact of Smad4 deficiency on LC antigen capture and migration *in vivo*. After painting the skin with FITC, draining LNs were harvested and analyzed by flow cytometry. There was no significant difference in the frequency of migrated FITC^+^ LCs (CD45^+^ MHCII^+^ Langerin^+^ EpCAM^+^ FITC^+^) between hSmad4KO and WT mice (Figure 2F). Therefore, Smad4 is not essential for either LC phagocytosis or migration in both *in vivo* and *in vitro* models. These findings are consistent with the observations in hSmad2KO mice.

### Smad2/4 Pathway Is Not Required in LC Development and Function

**Figure 3:**
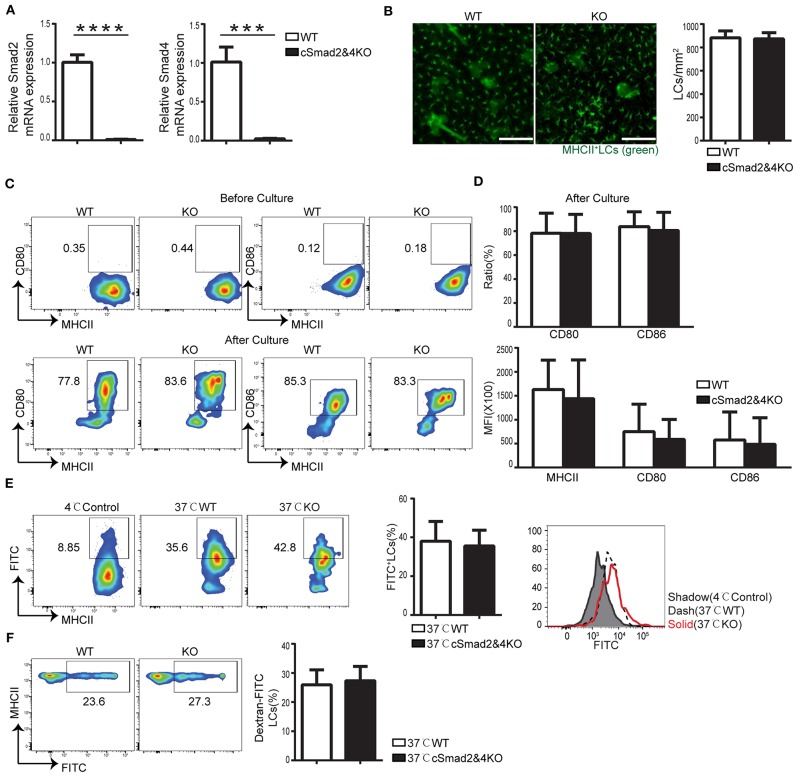
Smad2/4 signaling is not essential for LC homeostasis, maturation, phagocytosis, and migration. In cSmad2&4KO and WT mice, **(A)** Expression of Smad2 and Smad4 in sorted LCs by qRT-PCR (*n* = 6, ***P = 0.0009, ****P < 0.0001). **(B)** Epidermal sheets at 5 weeks old were stained with anti-MHCII (green). Scale bar = 100 μm, original magnification X10 (*n* = 6, *P* = 0.8484). **(C,D)** Flow cytometry analyses of LCs before and after 72 h of *in vitro* culture, the frequency of MHCII, CD80 and CD86 **(C)** and their expression **(D)** (*n* = 6–18, *P* > 0.5) were shown. **(E)** The frequency of FITC^+^ LCs after incubation with Dextran-FITC for 45 min at 37 or 4°C (as control) and FITC MFI in live LCs were shown (*n* = 6, *P* = 0.7591). **(F)** The frequency of FITC^+^ LCs in pre-gated migratory LCs in LNs (*n* = 7, *P* = 0.7301). Data were shown as mean ± SD.

To exclude the possibility of redundancy among Smad2, Smad3, and Smad4, we attempted to generate Csf1r^Cre^-induced conditional Smad2&3 double KO, Smad3&4 double KO, and triple KO mice; unfortunately, we were unsuccessful in making them so far. However, we were able to make Csf1r^Cre^ Smad2^fl/fl^ Smad4^fl/fl^ mice (cSmad2&4KO) that had a simultaneous deletion of both Smad2 and Smad4. The conditional deletion of Smad2 and Smad4 on LCs was confirmed by qRT-PCR (Figure 3A). Immunohistochemical staining of the epidermal sheets showed no significant difference in the number of MHCII^+^ LCs between cSmad2&4KO and WT mice (Figure 3B). In addition, FACS analyses further showed that there was no significant difference in the expression of MHCII, CD80, or CD86 in LCs between cSmad2&4KO mice and WT littermates before and after 72 h of culture (Figures 3C,D). Next, we evaluated LC antigen uptake and migration *in vitro* and *in vivo*, and observed no difference between them (Figure 3E,F). This was consistent with the results from individual hSmad2KO and hSmad4KO mice, further suggesting that the Smad2/4 pathway is not required in LC development at steady state. Given that Smad3 is not required for LCs (16), our data highly suggests that the Smad2/3/4 pathway is dispensable for LC maintenance, maturation, antigen uptake, and migration during the steady state.

### Smad2/4 Signaling Pathway Is Critical for “Long-Term” LC Repopulation During Inflammation

**Figure 4:**
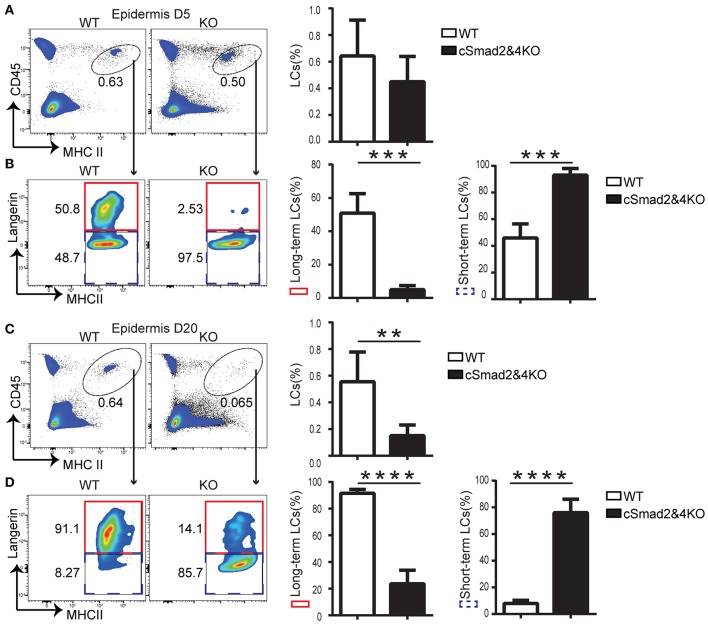
Smad2/4 signaling is required for “long-term” LC repopulation. **(A,B)** Smad2/4 signaling pathway is not required for “short-term” LC repopulation. **(A)** The frequency of LCs at day 5 (D5) after UV treatment (*n* = 9, *P* = 0.31); **(B)** The frequency of peripheral monocytes-derived “short-term” LCs (blue dashed line) and bone marrow-derived “long-term” LCs (red solid line) on pre-gated live LCs (*n* = 9, ***P < 0.0002). **(C,D)** Smad2/4 signaling pathway is required for “long-term” LC repopulation. **(C)** The frequency of LCs at day 20 (D20) after UV treatment (*n* = 12, **P = 0.0019); **(D)** The frequency of peripheral monocytes-derived “short-term” LCs (blue dashed line) and bone marrow-derived “long-term” LCs (red solid line) on pre-gated LCs (*n* = 12, ****P < 0.0001). Data were shown as mean ± SD.

Two waves of LC repopulation exist in the skin under inflammation conditions, including “short-term” LCs, which are derived from peripheral blood Gr-1^hi^ monocytes and express low levels of Langerin, and “long-term” LCs, which are derived from BM and express high levels of Langerin (8). Previous studies established a reliable mouse model treated with UVC to study LC regeneration under inflammation conditions, and uncovered that inhibition of differentiation and DNA binding 2 (ID2) was involved in “long-term” LC repopulation but not in “short-term” LC repopulation (8). To explore the role of the Smad2/4 signaling pathway in LC repopulation, we depleted epidermal LCs in cSmad2&4KO and WT mice by UVC treatment (8). The conditional deletion of Smad2 and Smad4 on BM-derived macrophages was confirmed by qRT-PCR (Figure S1), suggesting a specific myeloid cell lineage deletion. In addition, lack of Smad2/4 significantly blocked lung macrophage development in the embryonic and after birth, indirectly indicating that Smad2/4 was functionally deleted in the myeloid lineage in cSmad2&4KO mice (unpublished data). We next compared the ratio of “short-term” LCs (CD45^+^ MHCII^+^ Langerin^−^) and “long-term” LCs (CD45^+^ MHCII^+^ Langerin^+^) between KO and WT littermates at day 5 and day 20 post-UVC treatment. As expected, UVC treament effectively depleted epidermal LCs and only about 10% epidermal LCs left at day 3 in WT mice post-treatment (Figure S2). As shown in Figure 4A, there was no significant difference in the frequency of replenished epidermal LCs between cSmad2&4KO mice and WT controls at day 5. However, more than 95% of the LCs in cSmad2&4KO mice were Langerin^−^ “short-term” LCs and a few were Langerin^+^ “long-term” LCs, while about 50% of the LCs in WT mice were Langerin^−^ “short-term” LCs and about 50% were Langerin^+^ “long-term” LCs (Figure 4B). Whereas, a significant reduction in the frequency of replenished epidermal LCs in cSmad2&4KO mice compared to WT controls at day 20 was observed (Figure 4C), most of the LCs from cSmad2&4KO mice were Langerin^−^ “short-term” LCs (>80%). In contrast, very few LCs in WT mice were Langerin^−^ “short-term” LCs (<10%) (Figure 4D). We also analyzed the LCs in skin drain LNs at day 5 and day 20 post-UVC treatment, as showed in Figure S3, there was no significant difference on LC frequency and number between KO and WT mice. Long-term LCs are discriminated from short-term LCs by more abundant expression of EpCAM and CD24 on long-term LCs (8). As shown in Figure S4, the expression of EpCAM^+^ and CD24^+^ on “long-term” Langerin^+^ LCs were dramatically reduced in Csf1r^Cre^ Smad2^fl/fl^ (cSmad2KO) mice at day 5 post-UVC treatment, while the expression of EpCAM^+^ and CD24^+^ on short-term Langerin^−^ LCs were comparable between cSmad2KO and WT mice. Overall, the Smad2/4 signaling pathway is not required for the first wave of “short-term” LC repopulation derived from peripheral monocytes, but is required for the second wave of “long-term” repopulation from BM-derived progenitors.

## Discussion

TGFβ1 plays a central role in immune system development through various signaling pathways, which includes the canonical Smad2/3/4 pathway and non-canonical Smad-independent pathways (25). TGFβ1 is a crucial regulator of LC homeostasis and function after birth. Mice that lack TGFβ1, TGFβR1, or TGFβR2 exhibit a profound reduction in epidermal LCs (9, 10, 20). However, the specific underlying roles of TGFβ1 signaling in LC development and function remain unclear. Our previous research has shown that the TGFβ/Smad3 signaling pathway does not impact LC function or homeostasis (16). To further explore the role of TGFβ/Smad signaling pathways in LC maintenance and function, in this study, we first generated mice with LC-specific conditional deletion of Smad2 or Smad4, and found that LC-specific deletion of Smad2, Smad4, or both Smad2 and Smad4 had no significant effect on epidermal LC homeostasis during steady state. In addition, our preliminary data also suggests that the Smad2/4 pathway is not required for LC embryonic development as well as newborn pups either (unpublished data). Previous studies have shown that TGFβ1 regulates LC maturation and migration (19, 20). The inhibition of TGFβ1 and its signaling pathways can trigger LCs to spontaneously mature and migrate to skin-draining LNs (19, 20). No significant difference was observed in LC maturation and migration between KO mice and WT controls during *in vivo* assays. Furthermore, there was no significant difference in the expression of MHCII, CD80, or CD86 between KO and WT mice upon *in vitro* stimulation. Taken together with our previous results studying Smad3 (16), our current data suggests that canonical TGFβ/Smad2/3/4 signaling pathways are not required for maintaining epidermal LC homeostasis and immaturity at the steady state.

TGFβ receptors are known to also mediate a variety of non-Smad signaling pathways, which include mitogen-activated protein kinase (MAPK), extracellular signal-regulated kinases (Erks), c-Jun amino terminal kinase (JNK), p38 MAPK, IκB kinase (IKK), phosphatidylinositol-3kinase (PI3K), Akt, and Rho family GTPases (26). Recent research has shown that the PI3K/Akt/mTOR pathway plays an important role in DC development and function. The regulatory protein mTORC1 can stimulate PI3K, which leads to downstream activation of Akt and PI3K. Prior research has shown that the conditional deletion of Raptor, a component of the mTORC1 complex, leads to the progressive loss of epidermal LCs (27). However, the loss of mTORC1 activity did not affect the initial epidermal seeding with LCs in this study (27), which differs from the phenotype of TGFβ1 or its receptor loss in LCs (19, 20). Young mice with Raptor deficiency have increased LC migration out of the skin, resulting in increased migratory LC frequency inside skin-draining LNs, which is similar to the phenotype in TGFβ1 or its receptor loss in LCs (19, 20). Therefore, the loss of epidermal LCs in mTORC1KO mice is due to increased migration out of the skin, which only partially reflects the phenotype of TGFβ1 or its receptor loss in LCs. The role of the TGFβ/PI3K/Akt pathway in LC homeostasis still needs to be clarified in future research. Activated TGFβ receptors are also known to interact with TRAF6 and TGFβ-activated kinase 1 (TAK1). TAK1 activates several downstream kinases, such as JNK, p38 MAPK, and IKK. Interestingly, TAK1 is an essential regulator of DC homeostasis and function (28). Therefore, it would be very interesting to further investigate the role of TGFβ/TAK1/JNK/MAPK pathways in LC homeostasis and function in the future.

Like macrophages, under stress or inflammatory conditions, LCs may be generated from monocytes and BM-derived progenitors (29). There are two waves of LCs that repopulate the epidermis following UV-induced inflammation (8). The first wave involves Langerin^−^ “short-term” LCs derived from Gr-1^hi^ monocytes and is detected 5 days post-irradiation, which persists for up to 3 weeks; the second wave involves Langerin^+^ “long-term” LCs derived from undefined precursors of BM, which later become the overwhelming majority LC population (8). TGFβ1 has been found to regulate BM-derived LC repopulation via interaction with the inhibitor of differentiation and DNA binding 2 (ID2) (8). In addition, TGFβ1 has been found to regulate BM-derived LC repopulation via interaction with PU.1 in a RUNX-3-depedent pathway (30). Previous studies indicate that hLangerin^Cre^ reporter mice only mapped well-developed epidermal LCs and migrated LCs in skin-draining LN exclusively, without influencing other cell lineages including Langerin^+^CD8^+^ DCs (9, 19). Thus, hLangerin^Cre^ could not mediate gene deletion in the Langerin^−^ short-term LCs. Csf1r^Cre^ has been used to induce the gene deletion in the LCs as well as in the myeloid lineage cells, including monocytes and BM-derived macrophages (4). Thus, to investigate Smad pathways in LC repopulation under inflammatory conditions, we generated the myeloid lineage Smad2&4 KO mice mediated by Csf1r^Cre^ rather than by hLangerin^Cre^. In this study, we analyzed the frequency of repopulated LCs at day 5 and day 20 post-UV treatment using flow cytometry. We found that the conditional deletion of Smad2 and Smad4 together dramatically blocked the differentiation of MHCII^+^ Langerin^+^ “long-term” LCs during the inflammatory state, but had no significant effect on monocyte-derived MHCII^+^ Langerin^−^ “short-term” LCs. Since Smad2 must activate Smad4 to regulate the Smad downstream pathway, as expected, the deletion of Smad2 in LCs also displayed the interruption of long-term LC repopulation and the lower expression of EpCAM and CD24 on the long-term LCs, while short-term LCs were unaffected. Ideally, immunohistochemical analysis of LCs in the epidermal sheets would be analyzed alongside flow cytometry, but the limited availability of harvestable skin makes parallel analysis technically difficult. However, given the severe defect on the number of LC repopulation in cSmad2&4KO mice by FACS analysis, our data strongly supports our conclusion that Smad4 pathway is required for long-term LC repopulation.

A recent study, using an allogeneic hematopoietic stem cell transplantation model where allo-reactive T cells directly target LCs to ask if and how the LC network is ultimately restored, reports that donor monocytes are the precursors of the long-term LCs that are detected 3 weeks after transferring (31). One possible explanation to the discrepancy is that that transient exposure to UV irradiation compared to the prolonged inflammation caused by allo-reactive T cells may trigger different mechanisms of LC repopulation in the skin. Ly6C^+^ monocytes are short-lived non-cycling cells, which can quickly repopulate to skin and differentiate into MHCII^+^ Langerin^−^ “short-term” LCs in UV-induced acute skin damage model, which are detected 5 days post-irradiation and only persist for up to 3 weeks. By contrast, in the stem cell transplant model, the allogeneic T cells are recruited to the epidermis over a period of weeks, resulting in prolonged immune pathology and inflammation. Under this condition, the monocytes can become long-term LC, contributing to the emerging LC network (31). It is possible that the precursors of long-term LCs in the transient exposure to UV irradiation might be Ly6C^−^ monocytes derived from BM, while short-term LCs, which needs to be further investigated in the future. In addition, the question that whether the Ly6C^+^ monocyte-derived “short-term” LCs is an intermediate form of “long-term”LCs also needs further investigation under acute inflammation.

Both transcription factors PU.1 and ID2 play key roles in LC replenishment during inflammation. TGFβ1 is found to regulate BM-derived LC repopulation via interaction with the ID2 (8) or via interaction with PU.1 in a RUNX 3-dependent pathway (30). Thus, it is very likely that a Smad2/4-dependent pathway regulates PU.1 or ID2 activity during LC repopulation, which is currently under our investigation.

In conclusion, our data indicate that TGFβ1 regulates LC homeostasis and repopulation in both Smad4-dependent and Smad4-independent pathways. The Smad4 pathway is not required for LC homeostasis, maturation, antigen uptake, or migration during steady-state. In contrast, the Smad4 pathway is required for BM-derived “long-term” LC activity during inflammation. Based on the critical roles of LCs in skin immunity and diseases, our findings may help to develop novel therapeutics that target the TGFβ1/Smad4 pathway in LC-related skin diseases.

## Data Availability Statement

All datasets generated for this study are included in the article/Supplementary Material.

## Ethics Statement

The animal study was reviewed and approved by Institutional Animal Care and Use Committee of Henry Ford Health System. Written informed consent was obtained from the owners for the participation of their animals in this study.

## Author Contributions

LH and G-HL performed most of the experiments. Q-SM, ZY, LZ, and DK conceived and designed the experiments. LH, QY, NP, and YX analyzed the data. SC, XW, and IU-I assisted with animal maintenance and genotyped mutant mice. LH, Q-SM, ZY, and LZ drafted the manuscript.

## Conflict of Interest

The authors declare that the research was conducted in the absence of any commercial or financial relationships that could be construed as a potential conflict of interest.
