# Supplementary material for: Smad2/4 Signaling Pathway Is Critical for Epidermal Langerhans Cell Repopulation Under Inflammatory Condition but Not Required for Their Homeostasis at Steady State
Source: Front Immunol. 2020 May 7;11:912. doi: 10.3389/fimmu.2020.00912 (PMC7221176; doi:10.3389/fimmu.2020.00912)
Supplement: Supplementary file 1 [file Data_Sheet_1.PDF]

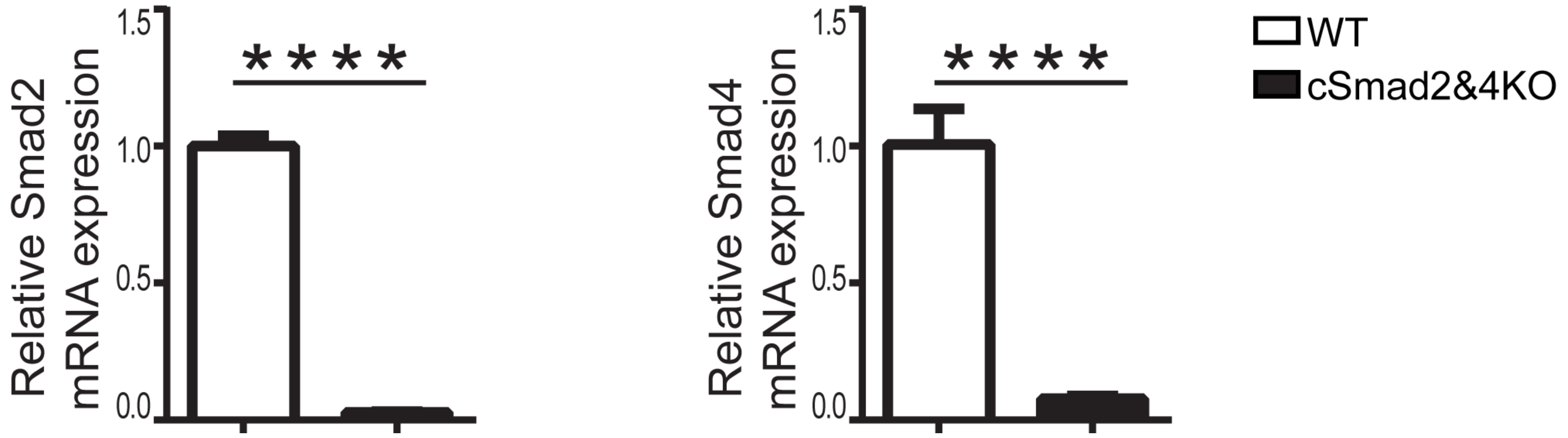

Fig S1. Expression of Smad2 and Smad4 in BM-derived macrophages. Isolated bone marrow cells from Csf1r^{Cre} Smad2&4$^{fl/fl}$ (cSmad2&4KO) mice were cultured in Iscove's Modification of Dulbecco's modified eagle medium (Cellgro) supplemented with 10% FBS (ATLANTA biologicals, Flowery Branch), 100 U/mL Penicillin, 100 µg/mL Streptomycin, and 30ng/mL macrophage colony-stimulating factor (Peprotech) for 1 week at 37ºC. Macrophages (CD11b^{+} F4/80^{+}) were sorted by BD AireII sorter and further verified for their purity (more than 98%). Total RNA from BM-derived macrophages was used for the expression of Smad2 and Smad4 by qRT-PCR (n=6, ***P=0.0009, ****P<0.0001).

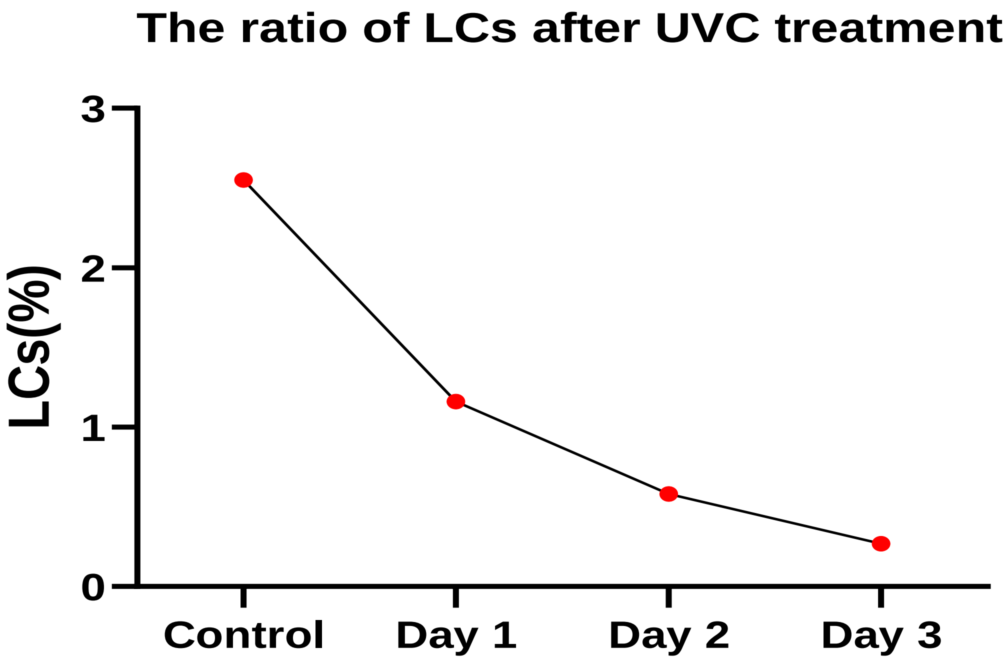

Figure S2. UVC induced LC repopulation. Mice were treated for 15 minutes with UVC. The back skin was taken from UVC-treated and control (non-UVC treated) day 1, day 2, day 3, for flow cytometric analysis. Up to 90% epidermal LCs were deleted at day 3.

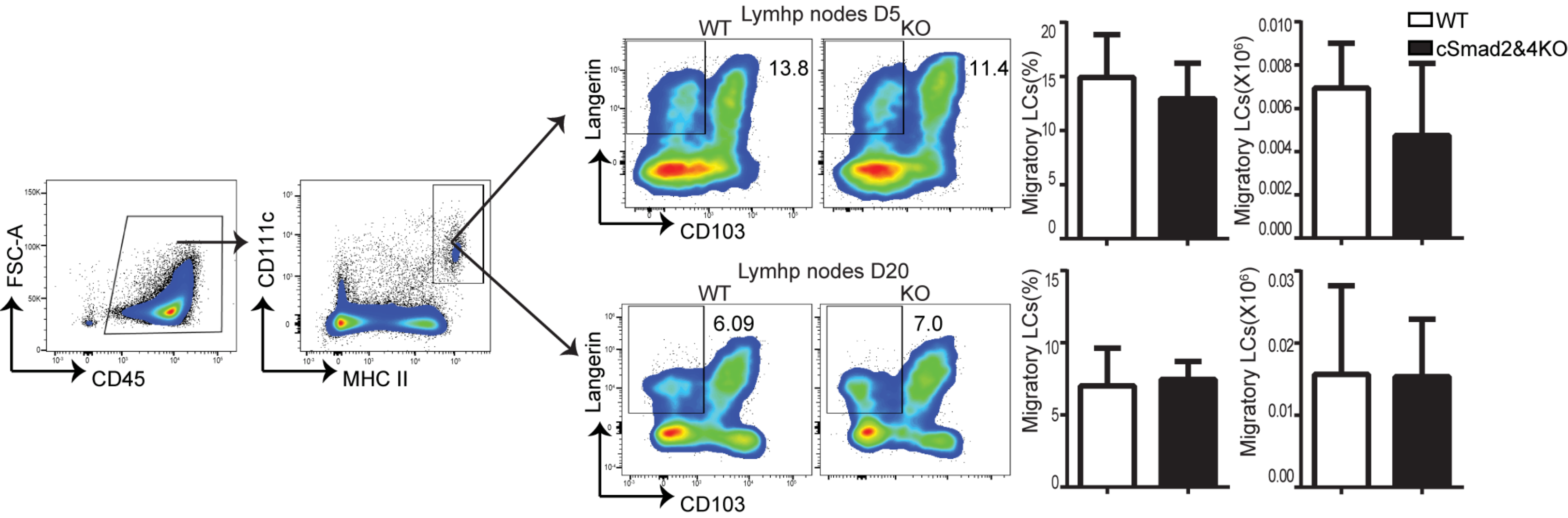

Fig S3. LCs in the draining lymph nodes during LC repopulation in cSmad2&4KO and WT mice. Draining lymph nodes (LNs) from cSmad2&4KO and WT littermates were collected for flow cytometry at day 5 and day 20 after UVC treatment. Density plots showing the gating scheme (left), frequency (middle) and bar plots (right) of LCs ($Langerin^{+}$ $CD103^{-}$ cluster pre-gated on $CD11^{+}$ $MHCII^{hi}$ cells) are shown (n=3, P >0.5). Data were shown as mean ± SD.

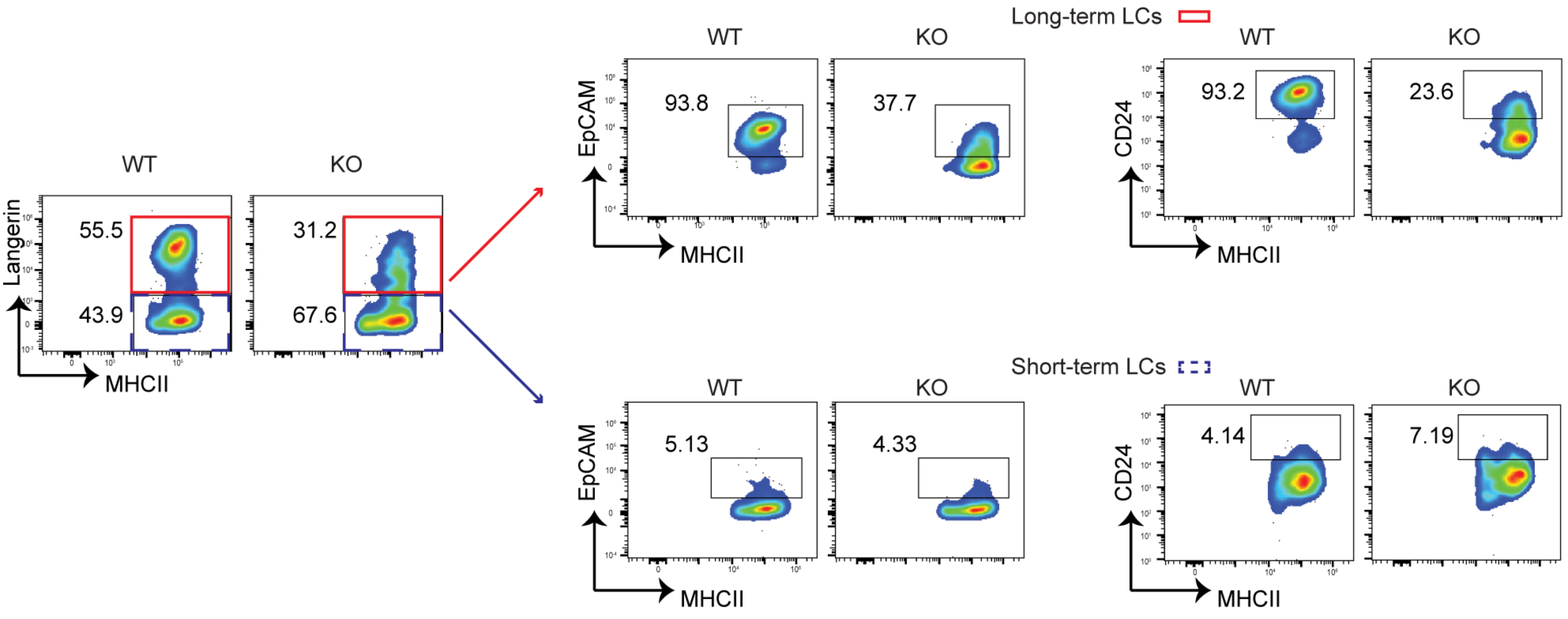

Fig S4. Smad2 is required for "long-term" LC development. Csf1r^{Cre} Smad2$^{fl/fl}$ (cSmad2KO) and WT littermates were treated with UVC for 15 minutes. UVC-treated back skin was harvested at day 5 post treatment to analyze epidermal LCs by FACS. Lack of Smad2 did not affect MHCII^{+} Langerin^{-} "short-term" LCs, but dramatically reduced the frequencies of MHCII^{+} Langerin^{+} "long-term" LCs (gated on CD45^{+} MHCII^{+} LCs). Furthermore, the frequencies of EpCAM^{+} and CD24^{+} population in "long-term" Langerin^{+} LCs were also dramatically reduced in cSmad2KO mice, while the frequencies of EpCAM^{+} and CD24^{+} population in Langerin^{-} short-term LCs were comparable between KO and WT mice.
